# Genomic and Evolutionary Insights into Australian Toxigenic Vibrio cholerae O1 Strains

**DOI:** 10.1128/spectrum.03617-22

**Published:** 2022-12-19

**Authors:** Murari Bhandari, Irani U. Rathnayake, Flavia Huygens, Son Nguyen, Brett Heron, Amy V. Jennison

**Affiliations:** a Centre for Immunology and Infection Control, Queensland University of Technology, Brisbane, Queensland, Australia; b Public Health Microbiology, Forensic and Scientific Services, Queensland Department of Health, Brisbane, Queensland, Australia; USGS, Eastern Ecological Science Center

**Keywords:** Australia, *Vibrio cholerae*, evolutionary microbiology, genomics, toxigenic, virulence determinants

## Abstract

Vibrio cholerae O1 is the causative agent of cholera, a severe diarrheal disease which can cause death if left untreated. In this study, a collection of clinical and environmental V. cholerae serogroup O1 isolates from Australia (1977 to 1987) (from local cases and cases acquired through international travel) and publicly available international isolates were characterized for genotypic features (virulence genes, mobile genetic elements [MGEs], and antimicrobial resistance gene profiles). Whole-genome sequencing (WGS) was used to investigate and compare the genetic relatedness between the 44 Australian and nine travel-associated isolates and the 60 publicly available international V. cholerae sequences representing pre-seventh-pandemic (pre-7PET) isolates and different waves of 7PET isolates. In this study, 36 (81%) Australian clinical and aquatic isolates harbored the cholera toxin-producing genes located in the CTX bacteriophage region. All the Australian environmental and clinical isolates lacked the seventh-pandemic virulence-associated genomic islands (VSP-I and -II). *In silico* multilocus sequence typing (MLST) classified all nine internationally acquired isolates as sequence type 69 (ST69), 36 clinical and aquatic isolates as ST70, and eight isolates from Australia as ST71. Most of the nontoxigenic clinical and aquatic isolates of ST71 had diverse genetic variations compared to ST70 Australian strains. The antimicrobial resistance-associated genes *gyrA*, *parC*, and *parE* had no mutations in all the environmental and clinical isolates from Australia. The SXT genetic element and class 1 integron gene sequences were not detected in Australian strains. Moreover, in this study, a Bayesian evolutionary study suggests that two distinct lineages of ST71 (new set of strains) and ST70 strains were prevalent around similar times in Australia, in ~1973 and 1969.

**IMPORTANCE** Australia has its own indigenous V. cholerae strains, both toxigenic and nontoxigenic, that are associated with disease. Exotic strains are also detected in Australian patients returning from overseas travel. The clinical and aquatic V. cholerae O1 toxin gene-positive isolates from Australia responsible for cases in 1977 to 1987 were linked to acquisition from Queensland waterways but until now had not been characterized genetically. It is important to determine the genetic relatedness of Australian strains to international strains to assist in understanding their origin. This is the first extensive study to provide sequences and genomic analysis focused on toxigenic O1 V. cholerae clinical and environmental strains from Australia and its possible evolutionary relationship with other publicly available pre-7PET and 7PET V. cholerae strains. It is important to understand the population genetics of Australian V. cholerae from a public health perspective to assist in devising control measures and management plans for reducing V. cholerae exposure in Australia, given previous Australian disease clusters.

## INTRODUCTION

Vibrio cholerae is a Gram-negative, small intestinal pathogen that causes the severe diarrheal disease cholera. Cholera is a major public health concern in many underdeveloped and developing countries, where millions of cases are reported each year and thousands of deaths occur globally (1). Cholera can occur endemically, leading to pandemics that cause major health and economic crises. To date, V. cholerae has been classified into more than 200 serogroups, of which cholera toxin-producing O1 and O139 are associated with major outbreaks and pandemics, while non-O1 and non-O139 isolates are mainly associated with moderate to severe human gastroenteritis (2). Serogroup O1 is further divided into two major biotypes, classical and El Tor, based on distinct genetic, biochemical, and phenotypic features (3, 4). V. cholerae can be an innocuous inhabitant of aquatic environments or can be pathogenic, mainly when water and food become the primary and secondary vehicles for cholera transmission in regions where cholera is endemic (5, 6). Transmission of cholera in regions where cholera is not endemic, such as Australia, is mainly associated with travelers that have acquired infections overseas or with consumption of raw or undercooked seafood imported from areas of endemicity (7, 8).

Since 1817, cholera cases reported globally at different times are classified into seven pandemics, and the seventh pandemic is still ongoing. The first six pandemics were linked to O1 classical strains and ceased around 1923. Before the seventh pandemic started in 1961, originating in Southeast Asia and caused by an El Tor strain (referred to as 7PET), there were some sporadic cholera cases caused by pre-7PET strains from 1923 to 1961 (9), including in Australia. The genomic approach applied to pandemic strains initially identified eight distinct phylogenetic lineages based on single nucleotide polymorphisms (SNPs), designated L1 to L8, with L1 and L3 to L6 representing the previous pandemics and L2 broadly representing three waves of seventh-pandemic El Tor cholera events (10). Later genomic studies from China and Africa further reported subclades within individual waves as well as several transmission events, namely, T1 to T12 from African countries, LAT-1 to LAT-3 from Latin American countries, and T13 from East Africa and Yemen. The genetic diversity, evolution, and global transmission events of seventh-pandemic O1 El Tor strains are discussed in detailed in several studies (10–14).

V. cholerae has many virulence genes and genomic islands that play a vital role in pathogenesis, among which cholera toxin (CT) and toxin-coregulated pilus (TCP) are major virulence factors (15, 16). The genes *ctxAB*, which encode cholera toxin, are part of the filamentous phage CTXϕ, and genes encoding TCP biosynthesis are located within *Vibrio* pathogenicity island 1 (VPI-1) (17). In recent years, type III and VI secretion systems (TTSS and T6SS) have been described as important virulence factors for the pathogenicity of O1 and non-O1 V. cholerae strains (18, 19). Self-transmissible mobile genetic elements (MGEs) that carry antimicrobial resistance (AMR) genes, such as the SXT element and class I and II integrons, are associated with the spread of AMR genes among V. cholerae and other bacteria (20, 21). It has been interesting to witness the diversity and the occurrence of these integrons and SXT elements, which are mainly found in 7PET lineages starting from the early 1990s from Asian countries where cholera is endemic (10, 11, 20, 22). The World Health Organization (WHO) recommends oral and intravenously administered solutions containing glucose, sodium chloride, potassium, and trisodium citrate for mild to moderate symptoms to avoid misuse of antibiotics that might have contributed to the emergence of antibiotic-resistant strains around the world (21).

Toxigenic cholera in Australia is associated with international acquisition leading to occasional outbreaks, such as a report of exotic strains in Sydney originating from food that was served on a flight from Bahrain (flight was from London to Sydney) in 1972 (23). In 1977, the first non-travel-related Australian toxigenic O1 cholera case was reported in southeastern Queensland, (24). Additional clinical cholera cases were described until 1987 from different cities in Queensland, with case exposures linked to Queensland river water systems, where subsequent testing identified toxigenic O1 V. cholerae isolates (25). However, there is limited understanding of Australian toxigenic V. cholerae O1 strains in Queensland waterways and whether these endemic strains still pose a risk of cholera outbreaks in Queensland. To date, these strains have not been well characterized or reported in the scientific literature. It is important to understand the population genetics of V. cholerae in Australia from a public health perspective.

This is the first genomic study to examine an extensive collection of unique toxigenic V. cholerae O1 isolates associated with clinical cases during local outbreaks, as well as isolates isolated from Queensland waterways between 1977 and 1987 and public health follow-ups and some exotic strains collected by the public health microbiology (PHM) laboratory of the Queensland Health Department. This study has extensively investigated the genotypic features of this unique data set and found two distinct lineages of V. cholerae pre-7PET (ST70 and ST71, a new cluster from Australia) using whole-genome sequencing (WGS) and bioinformatic analysis. Furthermore, our Bayesian analysis illustrates that the transmission of these Australian lineage strains’ ancestors into Australia or nearby regions occurred around 1955 (1929 to 1970) and that they dispersed around different cities in Queensland around the 1970s and may be due to natural disasters occurring at similar times.

## RESULTS

### Local epidemiology of Australian V. cholerae strains.

Since the occurrence of a cholera case in Queensland in 1977, public health investigations have led to an extensive collection of clinical isolates as well as aquatic isolates from Queensland that are epidemiologically associated with each disease episode. This collection also includes several other water samples from various waterbeds throughout Queensland, including river water, dam water, creek water, a sewerage plant effluent, fish and water storage tanks. Table 1 provides a list of locally acquired Queensland cholera cases since 1977 and possibly linked aquatic strains. Seven additional aquatic strains—B3673, v1, 8732_1, P412_1, P369_1, 7975_1, and v98_78—were collected from the Brisbane River, sewerage plants, and fish that were part of the surveillance, as shown in Data Set S1 in the supplemental material.

**TABLE 1:** Australian cholera cases caused by O1 toxigenic and nontoxigenic Inaba/Ogawa and river-linked aquatic V. cholerae strains in Queensland, Australia (55)

Mo/yr of isolation	Patient location (Queensland, Australia)	Clinical sample identification(s) (*n* = 15)	Possible source of infection	Aquatic sample identifications (*n* = 19) and surveillance (*n* = 7)^*a*^
Feb 1977	Beenleigh	4287_St, Si_B, Si_F	Albert and Logan Rivers	v6166, v4662, v4681, v4723, B17751_78, LA236, B5617_80, MV_1000_01
Feb 1980	Ipswich	B2166_80, B2592_80	Logan River	Fish_18, LA254
Jan 1983	Rockhampton	v0796, v0797, v0798, v0799	Fitzroy River	478_1, 6905_10_3_2, 431_C_2, v9840, v8479
Mar 1983	Beaudesert	3411_1, 3411_3	Logan River	340183100_1_1
Feb 1983	Mount Morgan	1633_1, 1633_2, 1633_3	Therese Creek	8821_2, v8823
Mar 1984	Townsville	8908_84	Burdekin and Clarke Rivers	5753_1

a See Data Set S1. Strains were collected from the Brisbane River, sewerage plants, and fish as part of a surveillance effort.

**FIG 1:**
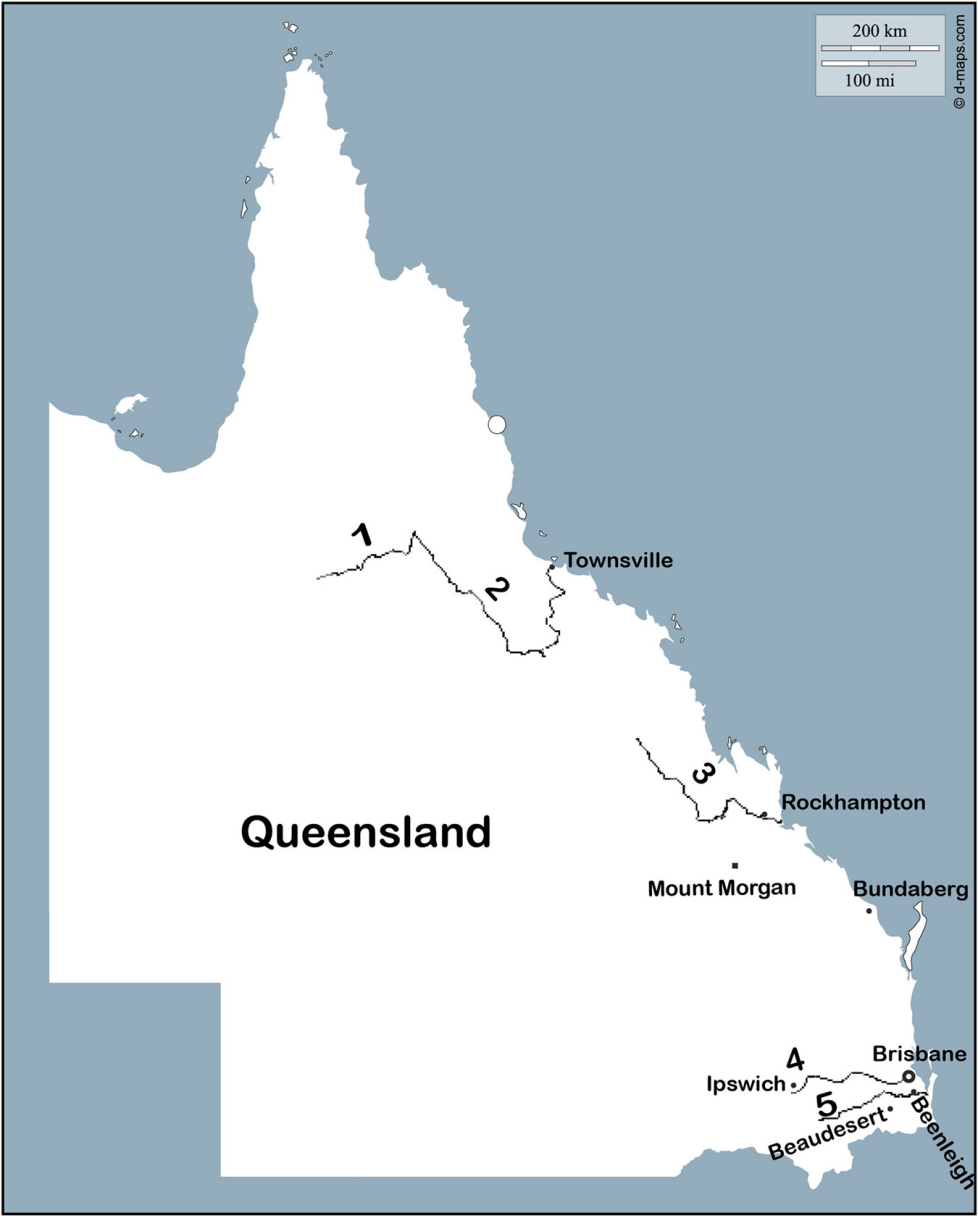
Rivers and locations associated with cholera cases in Queensland from 1977 to 1987 (55). The numbers 1 to 5 correspond to the Clarke River, Burdekin River, Fitzroy River, Brisbane River, and Logan River, respectively.

All of the clinical cases from Table 1 were reported during summer (January and February) and autumn (March) months from Queensland regions and were associated with waterways based on epidemiological studies (Fig. 1).

### Genetic classification of Australian and travel-associated V. cholerae strains.

**FIG 2:**
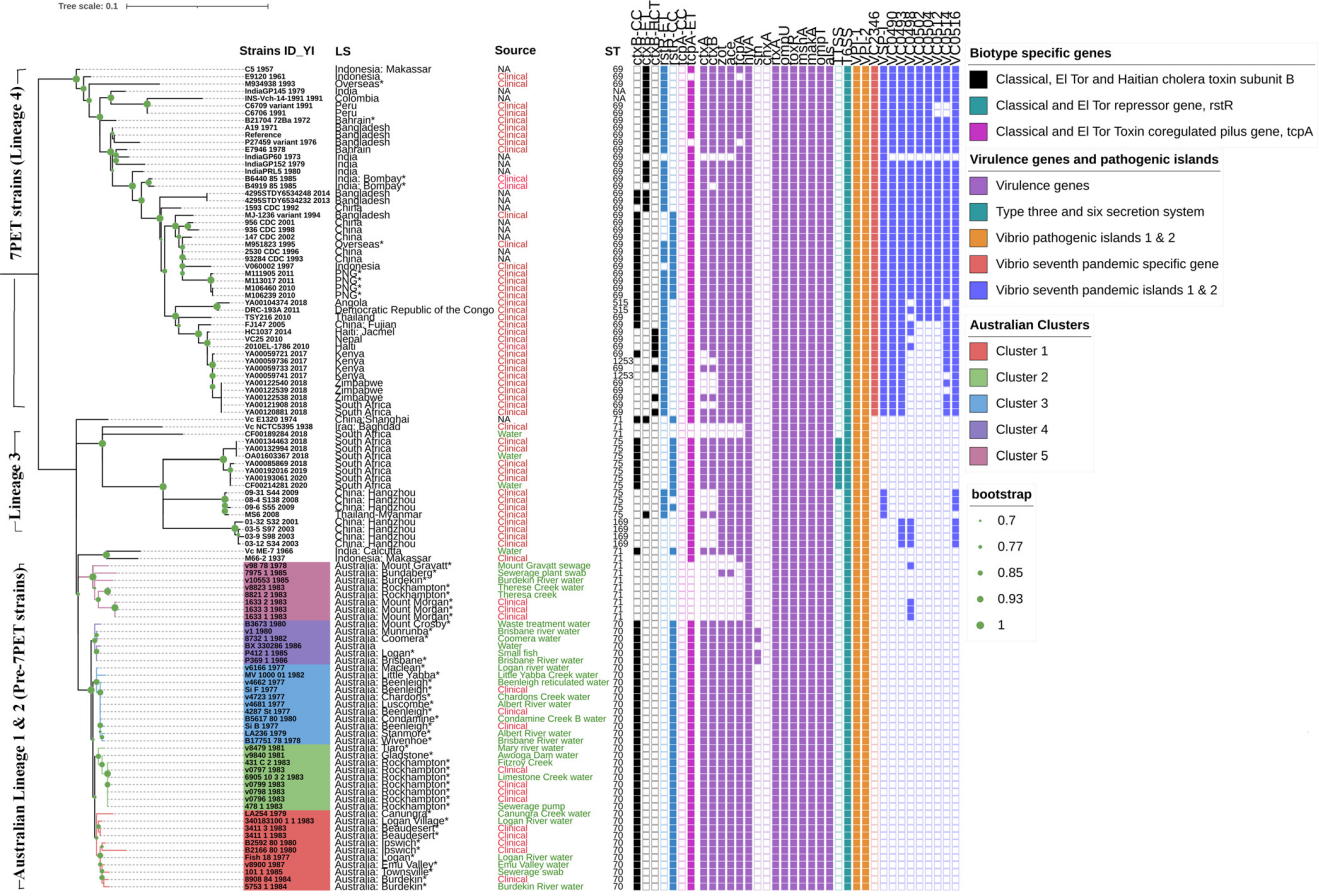
Maximum-likelihood phylogenetic tree of the pre-seventh- and seventh-pandemic strains of V. cholerae based on SNPs across the whole core genome, excluding likely recombination events and prophage regions. V. cholerae N16961 is used as a reference. Strain identities (IDs) with year of isolation, location of strains, biotype-specific characteristic features, virulence genes, VPI-1 and -2, seventh-pandemic-specific region, and VSP-I and -II profiles were generated using Interactive Tree Of Life (iTOL) (https://itol.embl.de/) and are represented with colored boxes for presence and white boxes for absence. Strains sequenced in this study are indicated with an asterisk. Based on SNP analysis, five clusters are shown in different colors for the Australian strains.

Among 53 sequences, nine clinical isolates were associated with overseas travel, 15 isolates were from Australian cases, and 29 were Australian aquatic strains. All the sequenced V. cholerae strains belonged to the O1 serogroup; 13 and 28 were serotyped as Ogawa and Inaba, respectively. Sequence types (STs) of all 53 strains were determined by multilocus sequence typing (MLST). All nine travel-associated isolates were ST69, corresponding to the 7PET lineage (Fig. 2 and Data Set S1). Of the Australian sequences, eight were ST71 and the remaining 36 were ST70, belonging to lineages distinct from 7PET (Fig. 2 and Data Set S1). We further classified these strains using Cholerafinder (https://cge.food.dtu.dk/services/CholeraeFinder/). This revealed that eight ST71 isolates from Australia were nontoxigenic, while 36/44 (81%) ST70 clinical and aquatic isolates from Australia were *ctxB* genotype 2, carried the El Tor type toxin-coregulated pilus gene (*tcpA*), and had the classical type of the repetitive sequence transcriptional repressor (*rstR*). The genotypes of the nine travel-associated strains varied considerably (Table 2).

**TABLE 2:** Genetic classification of Australian and travel-associated V. cholerae strains

Country of origin	Presence of biotype-specific genotype^*a*^							*ctxB* genotype^*b*^
	*ctxB*-Aus	*ctxB*-CC	*ctxB*-ET	*rstR*-ET	*rstR*-CC	*tcpA*-CC	*tcpA*-ET	
Australia (*n* = 36)	+				+		+	2
India, Bahrain, and overseas (unknown) (*n* = 3)			+	+			+	3
Papua New Guinea and overseas (unknown) (M951823) (*n* = 5)		+		+	+		+	1
India (*n* = 1)				+			+	NA

a +, presence; Aus, Australia; ET, El Tor; CC, classical.

b NA, not applicable.

### ST- and SNP-based phylogenetic analysis.

**FIG 3:**
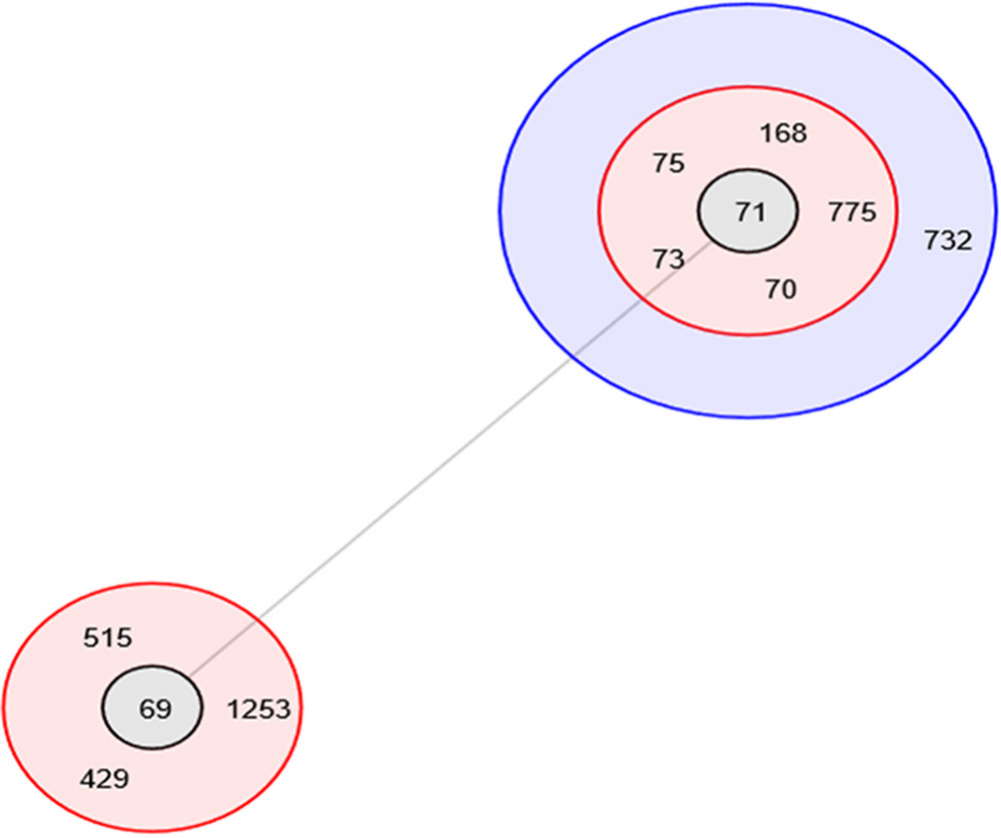
BURST analysis of sequence type 70 and its closest STs having five or more matches in their allelic profiles. A series of concentric different colored circles surrounding possible ancestral types (AT), shown in gray. STs in the red circles represent single-locus variants and blue represents double-locus variants with respect to the relevant AT (ST69 and ST71).

BURST analysis was performed to explore relatedness of the common Australian toxigenic V. cholerae sequence type ST70 to other publicly available closely related sequence types. ST71 and ST69 are single-locus variants (SLV) of ST70 (Fig. 3). ST70 strains are closely related to 7PET ST69 and pre-7PET ST71, with only one allele difference in the *pyrC* gene. This analysis also recognized ST71 as a potential ancestral type (AT) for ST70 (Fig. 3).

The PubMLST database (https://pubmlst.org/) was used to establish some context around ST70 strains and other closely related STs and their association with geographical regions. All four ST70 strains listed in the PubMLST isolate database were Australian, and three of the six ST71 strains were from Asia (China, India, and Indonesia), followed by one each from Iraq, Egypt, and South Africa. Among the 795 ST69 isolates in the PubMLST database, the majority were from South Asian and African countries, whereas of the 11 ST73 strains, three were from Bangladesh and India. Of the 43 ST75 strains, 12 were from China and Russia.

**FIG 4:**
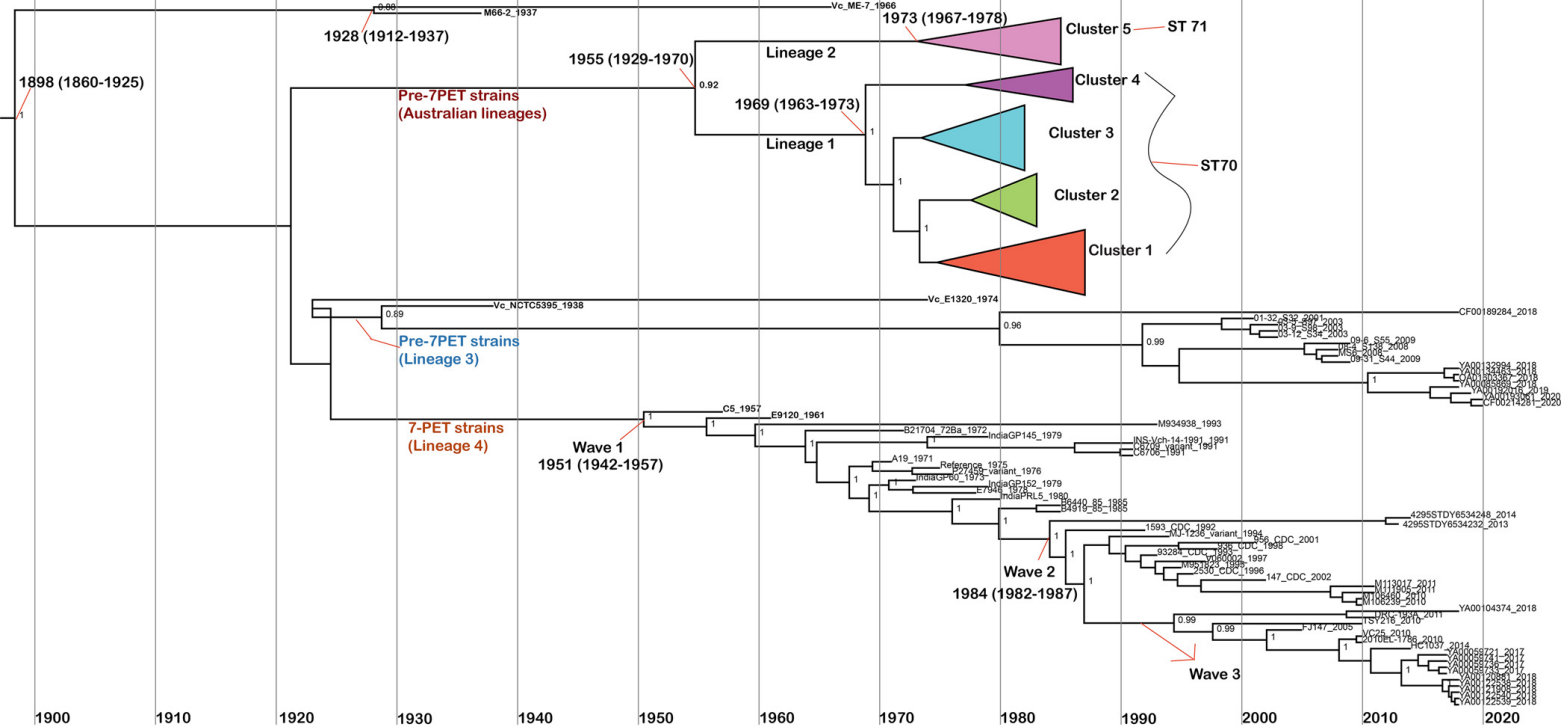
Maximum clade credibility tree generated from BEAST analysis. Node heights are the median values obtained from the BEAST analysis. Outlined dates are the median values predicted by BEAST, with the date range representing a 95% confidence interval for the estimate. The node values represent the credibility of the tree generated by BEAST. The dates shown are for the MRCAs of the O1 EL Tor lineage representing pre-seventh-pandemic and 7PET V. cholerae strains.

A maximum-likelihood tree was constructed based on genome-wide SNPs of all sequenced strains in this study with respect to reference strain N16961 and international sequences downloaded from publicly available databases (NCBI and EnteroBase). In this study, four distinct lineages were observed based on core SNP difference analysis excluding the recombination events of V. cholerae O1 El Tor strains isolated at different time points, sources, and locations. The first two lineages of Australian strains and a third lineage from Asia and South Africa correspond to pre-7PET, and lineage 4 corresponds to 7PET. The Australian cases and Queensland aquatic V. cholerae O1 El Tor strains of ST70 and ST71 causing disease clusters in different Queensland regions appear to have the same ancestral origin (Fig. 2 and 4). Within these Australian lineages, five different clusters were evident. Strains of Australian lineage 1 (ST70) formed multiple clusters, numbered 1 to 4, within the same lineage of strains, whereas strains of Australian lineage 2 (ST71) formed a single cluster, designated cluster 5 (Fig. 2 and 4).

Cluster 1 (≤17 SNP differences within the cluster) contains the sequences of isolates from Australian O1 toxigenic cholera cases in 1980, 1983, and 1984, which occurred in three different regions in Queensland, namely, Beaudesert, Ipswich, and Burdekin, and sequences from isolates from the Logan River, Burdekin River, a sewerage pump swab, and Emu Valley water. Notably, Australian clinical strain 3411_1_1983 showed only five SNPs compared to the Logan River water strain 340183100_1_1_1983, indicating the potential source for the case (Fig. 2 and Data Set S2). Cluster 2 comprises sequences from both locally acquired clinical cases and aquatic isolates. One clinical isolate, v0798_1983, differs by only 3 SNPs from two aquatic isolates (431_C_2_1983 and 6905_10_3_2_1983) collected from geographically close locations (Fig. 2 and Data Set S2). Cluster 3 represents the first few reported Australian O1 toxigenic cholera cases in 1977, Si_B, Si_F, and 4287_St, and these strains had only 4 to 11 SNPs compared to strains v4681_1977, v4723_1977, and v4662_1977, isolated from the nearby Albert River, Chardons Creek, and Beenleigh reticulated water (Fig. 2 and Data Set S2). Cluster 4 represents aquatic isolates (≤24 SNP differences within the cluster) from Queensland water sites, including a waste treatment plant and southeastern Queensland river water (Fig. 2 and Data Set S2). Notably, a previously sequenced Australian water strain, BX_330286_1986, showed only 10 SNP differences with P369_1986 and P412_1_1985, which were isolated from the Brisbane River (Data Set S2). Cluster 5 (≤37 SNP differences among cluster 5 strains) consists of nontoxigenic clinical strains (1983) and Burdekin River, sewerage plant, and Therese Creek aquatic isolates (1978 to 1985) of ST71 that formed a distinct cluster (Fig. 2 and Data Set S2). Overall, there were 150 to 1,200 SNPs in the Australian cluster (Data Set S2).

Strikingly, pre-7PET international strains M66-2_1937 (nontoxigenic) and Vc_ME-7_1966 (toxigenic) of ST71 from Makassar, Indonesia, and Kolkata, India, showed ~2,000 SNPs relative to Australian ST70 and ST71 strains. However, these strains (M66-2_1937 and Vc_ME-7_1966) showed many more (~4,000 and ~11,000) SNPs relative to other 7PET and pre-7PET strains from elsewhere (Fig. 2 and Data Set S2).

### Distribution of virulence gene profiles and pathogenicity-associated genomic islands.

Of the 53 sequenced strains, all nine internationally acquired toxigenic isolates (7PET lineage) harbored 19 of the 21 examined virulence-associated genes and genomic islands (VPI-1 and -2 and VSP-I and -II), lacking the heat stable enterotoxin (*stn*) and cholix toxin (*chxA*) genes. However, these nine isolates had the seventh-pandemic-specific marker, the VC2346 gene, whereas all the strains from Australia lacked this gene (Fig. 2). Of the 44 Australian clinical and aquatic cluster 1, 2, 3, and 4 isolates, 36 (81%) were toxigenic and carried most of the virulence-associated genes (*ctxA*, *ctxB*, *zot*, *ace*, *ace*, *tcpA*, *hlyA*, *als*, *toxR*, *rtxA*, *ompU*, *ompT*, *mshA*, and *makA*), including T6SS and *Vibrio* pathogenicity islands VPI-1 and -2, and lacked the seventh-pandemic islands (VSP-I and -II) (Fig. 2). Four water and three clinical Australian isolates from cluster 5 lacked some of the major virulence genes, namely, *ctxB*, *ctxA*, *zot*, *ace*, and *tcpA*. Interestingly, strain 7975_1 (ST71), a sewerage plant isolate from cluster 5, had *zot* and *ace* genes, indicating a truncated CTX phage genome (Fig. 2). Only four toxigenic water isolates (8732_1, v1, P369_1, and P412_1) from cluster 4 carried the *stn* gene (Fig. 2). All the Australian strains lacked the cholix toxin gene (*chxA*) and TTSS. In general, the occurrence of *chxA* gene is prevalent among non-O1, non-O139 V. cholerae strains and not common among 7PET strains. The diverse virulence gene profiles of the international pre-7PET and 7PET strains are shown in Fig. 2.

### TcpA analysis.

Interestingly, all the Australian El Tor V. cholerae isolates showed no differences in their *tcpA* sequences compared to the pre-7PET strain M66-2, including all the other 7PET isolates.

### Antimicrobial resistance gene profiles, class 1 integrons, plasmids, and mobile genetic SXT elements.

**FIG 5:**
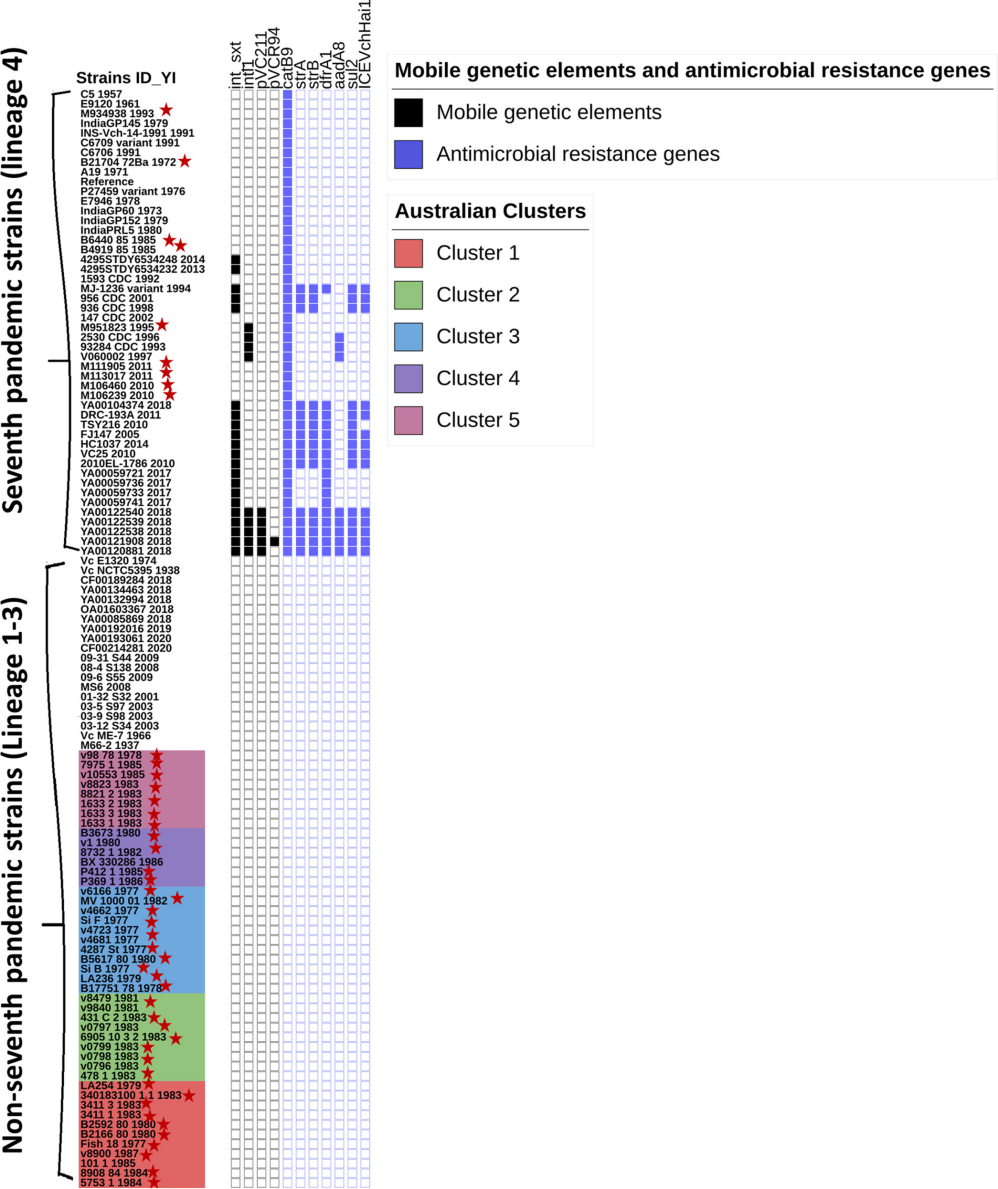
Presence-absence displays from iTOL annotation for the mobile genetic element and antimicrobial resistance gene profiles of V. cholerae strains sequenced in this study (marked with red stars) as well as publicly available international strains. All the Australian strains are shown in different-colored clusters at the bottom of the list. A colored box represents presence and a white box, absence.

An *in silico* analysis was performed using WGS data for the 53 sequenced strains to extract their antimicrobial resistance gene profiles, class 1 integrons, multidrug resistance (MDR) plasmids and MGEs. One of the travel-associated strains, M951823, had the class 1 integron (*intl1*) gene (Fig. 5). No SXT genetic elements, plasmids, or class 1 integron gene sequences were present in the Australian strains. All the Australian local aquatic and clinical isolates had the same profile of antimicrobial resistance-associated genes: DNA gyrase subunit A, DNA topoisomerase IV subunit A, and DNA topoisomerase IV subunit B (*gyrA*, *parC*, and *parE*). If these genes display nonsynonymous SNPs in the quinolone resistance-determining region (QRDR) (e.g., a SNP leading to GyrA_S831), this would lead to the resistance to quinolones. All the Australian strains showed no SNPs in the *gyrA*, *parC*, and *parE* genes compared to the 7PET reference strain N16961. The diverse MGEs and antimicrobial resistance-associated gene profiles of international strains together with other pre-7PET and 7PET strains are shown in Fig. 5.

### CTX and other prophage analysis among Australian and travel-associated V. cholerae strains.

The presence of prophages in the Australian and travel-associated strains and 10 additional publicly available international strains were determined using the online tool PHASTER. As shown in Data Set S3, different prophages with or without the CTX phage were observed as an intact, complete region in 84% (53/63) of the isolates. The CTX phage was identified in 81% (36/44) of the 12 clinical and 24 aquatic Australian isolates. In this study, interestingly, only Australian lineage 1, cluster 4, aquatic strains P412_1, P369_1, and Fish_18 had the CTX phage and an additional prophage. Australian aquatic strains P412_1 and P369_1 had CTX and *Vibrio* Martha12B12 prophage sequences, whereas Fish_18 had CTX and *Vibrio* K139 prophage sequences (Fig. 2 and Data Set S3). Seven Australian clinical and aquatic strains and one international strain (M66-2) did not have any completely intact prophage sequences.

To investigate the CTX phage genetic relatedness and differences between Australian and international strains, CTX phage sequences of these isolates were aligned to the V. cholerae reference strain N16961, and multiple-sequence-alignment analysis was used to visualize sequence variations.

From the multiple-sequence alignments, the Australian pre-7PET strains showed low numbers of SNPs within the CTX phage compared to seventh-pandemic typical and atypical El Tor international strains (Data Set S4). The genetic uniqueness of the CTX phage region of Australian clinical and aquatic V. cholerae strains were investigated based on SNPs and compared to the CTX phage region of 7PET strains (Data Set S4). Australian clinical and aquatic V. cholerae strains had only one SNP, in the *cep* gene at position C45G. Three SNPs were detected in a hypothetical region coding sequence: G336A, C726A, and A1166C (Table S1 in Data Set S4). Five SNPs in the *ace* gene at T18C, C39T, C102T, G219A and C270T (Table S2 in Data Set S4). In addition, *zot* with 15 SNPs, *ctxA* with six SNPs, and *ctxB* gene sequence with 3 SNPs at 115(C), 138(G), and 203(C) were carried by clinical and aquatic V. cholerae strains from Australia (Tables S3 and S4 in Data Set S4). Interestingly, Australian lineage 2, cluster 5, ST71 sewerage swab strain 7975_1_1985, isolated from Bundaberg, Queensland, showed a similar *ace* sequence with only one SNP at T219G and *zot* sequences with 13 SNPs (a component of the CTX phage region) relative to the 7PET strains (Data Set S4).

### Evolutionary history of Australian pre-seventh- and seventh-pandemic Vibrio cholerae.

A Bayesian analysis of core SNPs was used to estimate the dates for branching at the nodes. According to our genomic analysis, the date of the most recent common ancestor (MRCA) of the El Tor strains that includes pre-7PET (ST71), Australian (ST70 and ST71), and 7PET (ST69) Vibrio cholerae strains was 1898 (95% highest posterior density [HPD] interval, 1860 to 1925) (Fig. 4). In the case of Australian V. cholerae El Tor strains, it was observed that in 1955 (95% HPD interval, 1929 to 1970) the ancestor for Australian strains diverged into two distinct Australian lineages, 1 and 2, which then further subclustered into ST70 strains as cluster 1 to 4 strains and ST71 as cluster 5 strains (Fig. 2 and 4).

Notably, based on our Bayesian analysis, after World War I (1920 to 1930), the divergence of the ancestors of Australian ST70 and ST71 strains (lineages 1 and 2), other pre-7PET strains (ST71, ST75, and ST169) (lineage 3), and 7PET (lineage 4) strains occurred increasingly, resulting in distinct lineages leading to later outbreaks and pandemics. Remarkably, Iraq pre-7PET ST71, strain Vc_NCTC5395_1938 and other recently isolated clinical and environmental ST71, ST75, and ST169 epidemic lineage 3 strains from South Africa, Thailand, and China shared a MRCA and dated to 1929 (95% HPD interval, 1914 to 1937). Based on our study, there were multiple transmission events that occurred prior to outbreaks in South Africa caused by ST75 strains in 2018 to 2020 (26) and in China caused by ST169 strains in 2001 to 2009 (27) (Fig. 2 and 4).

The MRCA of Australian lineage 1 ST70 strains was dated to 1969 (95% HPD interval, 1963 to 1973) and that of Australian lineage 2 ST71 to 1973 (95% HPD interval, 1967 to 1978) (Fig. 4). The overall mutation rates of lineage 1, including clusters 1 to 4, and cluster 5 in lineage 2 were estimated as shown in Table 3 and Fig. 4.

**TABLE 3:** Mutation rates for Australian V. cholerae strains in five clusters within lineages 1 and 2

Australian lineage	Cluster	Mutation rate (substitutions/site/genome/yr)	95% HPD interval
1		2.71 × 10^−7^ (1.1)^*a*^	1.3 × 10^−8^–8.6 × 10^−7^
	1	3.1 × 10^−7^	1.3 × 10^−8^–8.6 × 10^−7^
	2	2.0 × 10^−7^	1.78 × 10^−8^–4.95 × 10^−7^
	3	3.6 × 10^−7^	1.85 × 10^−8^–9.8 × 10^−7^
	4	1.2 × 10^−7^	1.28 × 10^−8^–2.76 × 10^−7^
2	5	2.15 × 10^−7^ (0.9)^*a*^	2.27 × 10^−8^–4.77 × 10^−7^

a Substitutions per genome per year based on a reference genome of 4,047,835 bp.

## DISCUSSION

V. cholerae is an inhabitant of estuarine and coastal waters. In Australia, locally acquired cholera cases, caused by both nontoxigenic and toxigenic O1 V. cholerae isolates, have been linked to Queensland waterways. However, their genomic relatedness and ancestral origins were unknown.

This study is the first to carry out a large-scale genomic investigation of Queensland (Australian) V. cholerae O1 toxigenic isolates. It was interesting that both toxigenic and nontoxigenic O1 clusters contained clinical and aquatic isolates of similar genotypes. The presence of partial CTX phage regions (*zot* and *ace*) in Australian lineage 2, cluster 5, strain 7975_1 indicates loss of the CTX phage (mobile genetic element) in closely related (<37 SNPs) ST71 strains within the same cluster (cluster 5). Nontoxigenic ST71 strains are also less different from most other Australian clinical and aquatic ST70 (<1,200 SNPs) strains than international strains, indicating their ancestral relatedness. Consistent with other studies, our study also hypothesized that the evolution of Australian V. cholerae strains might have resulted from genetic exchanges between toxigenic and/or nontoxigenic V. cholerae strains and pre-7PET strains, based on the acquisition of virulence genes and SNP-based analysis (28).

Results from this investigation revealed that the most prevalent sequence type Australian V. cholerae O1 strains is ST70 (36/44 strains) along with eight ST71 strains. BURST analysis of the closest STs suggested that ST71 is the precursor of ST70 and ST69 (Fig. 3). ST71 strains have been isolated from Asia (China, India, and Indonesia), with no previously reported strains in Australia. The literature also notes that the pre-7PET strain M66-2 (Makassar, Indonesian, 1937) and 7PET strain N16961 (Bangladesh, 1975) are ST71 and ST69, respectively, and they are the closest to Australian ST70 strains, with a single-locus variant only based on MLST eBurst analysis (29). It is likely that Asian and Australian O1 El Tor strains share an ancestor; however, the evolutionary direction is unknown.

In a previous study by Safa et al., Australian aquatic isolates isolated between 1977 and 1986 lacked the seventh-pandemic islands, and this correlates with the current study of aquatic and clinical isolates from that time period (28). All the Australian clinical and aquatic isolates lacked seventh-pandemic islands, consistent with other pre-7PET isolates isolated elsewhere (Asia, Middle East, and South Africa). In this study, for the *ctxB* gene, all the Australian toxigenic O1 strains were classified as genotype 2 (Table 2), which is consistent with other studies based on the positions of amino acids: His(20), Gln(24), Asp(28), His(34), His(39), Leu(46), Lys(55), and Thr(68).

The distribution and occurrence of *Vibrio* prophages among serogroups are of interest. Studies have shown that bacteriophages play an important role in the evolution and virulence of many pathogens (15, 30, 31). The phage-biotyping scheme is used to distinguish between epidemic strains, to trace the transmission of the epidemic, and to identify the source of the outbreak based on the phage resistance phenotype and its genetic mutations (32). Initially, the temperate phage *Vibrio* K139, used in phage typing, was observed among V. cholerae O139 strains (India) and was later detected in V. cholerae O1 and non-O1, non-O139 strains (33–35). Even in the current study, aquatic and clinical strains from Australia and strains from India shared similar *Vibrio* CTX and *Vibrio* K139 phage profiles (Data Set S3), which is also indicative of the close relationship of Australian V. cholerae O1 strains to Asian strains. The evolution of the Australian CTX phage remains to be fully investigated.

According to a study by Duchêne et al., the evolutionary rates for bacteria has been estimated to be of approximately 1 × 10^−5^ to 1 × 10^−8^ nucleotide substitution per site per year (36). Similarly, in our study, all the Australian and international V. cholerae El Tor strains showed 1 × 10^−7^ nucleotide substitution per site per year. The extent of evolution varies among clinical and aquatic isolates. In contrast to the study by Mutreja et al. (10), which reported the mutation rate among V. cholerae El Tor isolates as 3.3 SNPs per year, aquatic isolates showed extensive diversity of >2% SNPs compared to clinical strains in another study (37). Furthermore, in our phylodynamic study, the mutation rate for Australian ST70 and ST71 strains was about 20-fold lower than that for the 7PET strains (2 to 3.24 substitutions per genome per year) at 0.12 substitution per genome per year, considering a reference genome size of 4,047,835 bp (95% HPD interval, 2.9 × 10^−9^ to 8.9 × 10^−8^). It is our understanding that lower mutation rates and the noncompetitive environment for Australian pre-7PET strains correlates with the inability of these strains to acquire virulence genes specific to seventh-pandemic strains to cause pandemics.

Based on our study, we propose that there were two possible separate transmission events for ST70 and ST71 of Australian V. cholerae isolates that were present in the Australian water or geographically colocated in nearby Queensland regions during similar times in the late 1960s to early 1970s, as shown by the MRCA dates in Fig. 4. Based on the genetic relationship among Australian strains with low numbers of SNPs (0 to 1,200 SNPs) compared to travel-associated and international strains, it is possible that the Australian strain ancestors were introduced into Australian waters or nearby regions sometime before they began to cause disease clusters and were subsequently transmitted during outbreak events or spread as a result of cyclones, floods, contaminated drinking water, or seafood or even by the excreta of infected persons arriving in different regions of Queensland from outbreak regions later on. Nontoxigenic O1 cluster 5 clinical strains (1633_1, 2 and 3) from Mount Morgan (coastal northern Queensland) are genetically closely related to nontoxigenic aquatic V. cholerae O1 strains from Rockhampton (Theresa Creek water), Bundaberg, and Mount Gravatt (sewerage plant), predominantly from coastal regions of Queensland. Interestingly, strain 98_78_1978, isolated from a Mount Gravatt sewerage plant, and other clinical and environmental strains from cluster 5 of same sequence type, ST71, have a common ancestor dated to 1973 (1967 to 1978). In addition, the similar genotypes of Australian lineage 1, cluster 1 to 4, clinical and aquatic strains have a common ancestor despite the geographical distance and source, supported by the possibility of uncommon transmission events, such as natural disaster events (Fig. 2 and 4). Coincidently, natural disasters such as Cyclone Althea (1971) (http://www.bom.gov.au/cyclone/history/althea.shtml) and Cyclone Emily (1972) (http://www.bom.gov.au/cyclone/history/emily.shtml), as well as the largescale Brisbane flood (1974) (http://www.bom.gov.au/qld/flood/fld_reports/brisbane_jan1974.pdf), all occurred in the 1970s in Queensland coastal regions. Based on our study, these natural disaster events may have contributed to the dissemination of ST70 and ST71 isolates across several Queensland regions in the early 1970s.

Moreover, according to our Bayesian analysis, the MRCA for all four distinct lineages of diverse pre-seventh-pandemic and 7PET strains had a high confidence level and was dated to 1898 (95% HPD interval, 1860 to 1925), which relates to the initial report of nonpathogenic Middle East El Tor strains in 1897 (38, 39). From 1920 to 1928, a massive diversification of El Tor strains occurred, giving rise to El Tor strains that are precursors of outbreak and 7PET-causing strains, as shown by the MRCA dates in Fig. 4.

The introduction of cholera from Mecca (Middle East) via Makassar by diseased humans (from Makassar or elsewhere), ballast water, or pilgrims (from Mecca; also, pilgrims might not be sick but can transmit the pathogen) into Australia has been hypothesized. As suggested by Hu et al., pathogenic strains from Mecca were spread to Makassar and Australia (39), as Makassar is relatively close to Australia geographically and was a major trade and fishing port. As previously documented, Australian trepang (dried sea cucumbers [*Holothuroidea*]) were sold in Makassar and shipped to China (40). Also, annual visits to the northern and western coasts of Australia by a fleet of boats from Makassar occurred during the 18th century. Also, annual visits to the north and west coasts of Australia by boats from Makassar, Indonesia occurred during the 1800s, ceasing in 1908, but fishing with landings has continued (39, 41). Interestingly, in our study, Australian and ST70 and ST71 strains and other pre-seventh-pandemic and 7PET strains share ancestors, with a MRCA date between 1920 and 1930, including two pre-7PET strains (M66-2_1937 and Vc_ME-7_1966) from Indonesia and India, respectively. Thus, because of the similar profiles based on SNPs, shared ancestral history, sequence type, and phage type of Australian strains and Asian strains, we also support the hypothesis of the transmission of pathogenic V. cholerae strains from Asia to Mecca, then to Makassar, and finally to Australia, as discussed above, based on our phylodynamic study. However, the possibility of direct transmission of pathogenic V. cholerae strains from Asia to Australia independently still remains. Due to the absence of the phylogenetically related precursor ST71 strains isolated soon after the diversification of El Tor (1920 to 1930) from Asia, this is less likely to happen.

Further studies are required to understand the evolutionary changes in Australian V. cholerae O1 strains isolated from the environment, as there have not been any outbreaks caused by imported strains since the 1980s, which could be indicative of well-managed waterways and sanitary systems in Australia. However, the risk of cholera and cholera-like disease still remains due to the consumption of raw and undercooked seafood and untreated water, given the previous local outbreak events and wide distribution of toxigenic V. cholerae O1 strains in Queensland waterways.

### Conclusion.

In the current study, WGS and bioinformatic tools were used to analyze historical Queensland clinical and aquatic V. cholerae O1 strains. We confirmed that all the clinical cases were genetically related to Queensland waterway isolates, providing evidence of their being responsible for human disease. In addition, all the Australian strains were determined to be unique pre-7PET ST70 strains and *ctxB* genotype 2 (Table 2), with only a few ST71 strains. Two separate transmission events might have occurred for Australian ST70 and ST71 isolates, which then circulated within Queensland due to natural disasters. Some of the internationally acquired strains showed atypical El Tor characteristic features from this *in silico* analysis. Findings from this study contribute to our understanding of the epidemiology and genetic characteristics, including virulence genes and antimicrobial resistance profiles, of Australian V. cholerae O1. This information can ultimately assist public health microbiology laboratories to apply control measures and undertake management of cholera in Australia.

## MATERIALS AND METHODS

### Selection of strains, DNA extraction, and whole-genome sequencing.

Vibrio cholerae O1 nontoxigenic and toxigenic strains from clinical and water samples sequenced in this study were collected from several rivers and household water tanks in cities/towns of Queensland (Fig. 1 and Data Set S1). In addition to the 15 clinical, 29 water, and nine exotic strains (all isolated in Australia), 60 publicly available international strains were also investigated in this study (Data Set S1).

DNA was extracted from isolates cultured overnight at 37°C on horse blood agar (Edwards Group Pty. Ltd.; no. 04059), using the QiaSymphony DSP DNA minikit (Qiagen) according to the manufacturer’s instructions. Libraries were prepared for sequencing using the Nextera XT kit (Illumina) and sequenced on the NextSeq500 platform using the NextSeq 500 mid-output kit (v2; 300 cycles) (Illumina) according to the manufacturer’s instructions. Sequence reads for the V. cholerae isolates were trimmed with Trimmomatic v0.36 (42) and quality checked by FastQC v0.11.5 and MultiQC v1.1 (43). Sequence reads that had >75% of the read length in the green zone of the mean quality score graph on FastQC (Q score, >28), that had an average read length of >120 bp, and that contributed to the majority of reads over 140 bp, according to the sequence length distribution graph, were selected. *De novo* assemblies were generated with the SPAdes assembler v3.12.0 (44); the quality of the assemblies was analyzed using QUAST 4.6.3, and annotation was done by using Prokka. The quality of assemblies was determined based on the contigs ≥500 bp in length, which had to be less than 500 in number, and the total length of the assembled contigs had to be similar (within 30%) to the expected genome median from National Center for Biotechnology Information (NCBI) genomes.

### Genotyping of Australian, travel-associated, and international V. cholerae isolates.

Genome assemblies of Queensland and travel-associated strains were analyzed by using the public database PubMLST using FASTA sequences to identify sequence types (ST) using the MLST scheme. BURST (based upon related sequence type) v1.1.9 in PubMLST (https://pubmlst.org/) is a clustering algorithm that uses MLST data to establish relationships within clonal complexes among similar allelic profile strains. In this analysis, five or more locus-matching profiles were selected to determine the relatedness between ST70 and other publicly available STs.

All the whole-genome sequenced assemblies (FASTA sequence) were submitted to the PHASTER online web interface (https://phaster.ca/) with default settings to detect the presence of Vibrio cholerae prophages. The results were tabulated to determine their relatedness and diversity.

### Virulence gene analysis of Australian, travel-associated, and international O1 El Tor V. cholerae isolates.

All the V. cholerae strains selected for this study were previously identified and characterized by using a combination of biochemical, serological, and molecular methods. Furthermore, in this study, whole-genome sequences (reads) of all the strains were characterized by using the bioinformatic tool Cholera Finder v1.0, as described at the Center for Genomic Epidemiology website (https://cge.food.dtu.dk/services/CholeraeFinder/). This tool uses BLAST as a search engine and was used to determine the presence of a species-specific gene (*ompW*), serogroup-specific genes (*rfbV* [O1] and *wbfZ* [O139]), biotype-specific genes (*ctxB*, *rstR*, and *tcpA*), a 7PET-specific gene (VC2346), collective virulence genes (*ctxA*, *ctxB*, *zot*, *ace*, *tcpA*, *hlyA*, *stn*, *chxA*, *rtxA*, *ompU*, *toxR*, *mshA*, *makA*, and *als* and TTSS and T6SS genes), and pathogenicity islands (VPI-1, VPI-2, VSP-I, and VSP-II) in all V. cholerae genomes with a threshold equal to 95% identity and 60% coverage, as previously described (45). For *ctxB* genotyping, the *ctxB* gene sequences were extracted from annotated strains and analyzed in Geneious (https://www.geneious.com/) using a multiple-sequence alignment tool, CLUSTALW (http://www.clustal.org/clustal2/). This was used to determine sequence similarities and uniqueness among Australian and international *ctxB* genes.

Antimicrobial resistance genes were detected using the ResFinder tool (https://cge.food.dtu.dk/services/ResFinder/) for all V. cholerae genomes with a threshold equal to 95% identity and 60% coverage, as previously described (45). The mobile genetic element (ICE-SXT), class 1 integron genes, plasmids, and mutations in the DNA gyrase gene (*gyrA*) and the DNA topoisomerase IV genes (*parC* and *parE*) were determined using MyDbFinder (https://cge.food.dtu.dk/services/CholeraeFinder/).

### Phylogenetic inference and Bayesian evolutionary analysis.

To perform phylogenetic analysis, 53 sequenced Australian and exotic strains, as well as 60 international genome sequences obtained from public databases (NCBI [https://www.ncbi.nlm.nih.gov] and EnteroBase [https://enterobase.warwick.ac.uk/species/vibrio/search_strains]) were used. Limited international strains were selected based on their geographic location and known genotypic features to cover the genomic waves described previously for 7PET strains (10). Variants were called using Snippy version 4.3.6 (https://github.com/tseemann/snippy) using the seventh-pandemic V. cholerae O1 El Tor N16961 genome as a reference (GenBank accession numbers NZ_CP028827.1 and NZ_CP028828.1). The single-nucleotide variants (SNVs) in recombinogenic regions were detected using Gubbins version 2.3.4 (46), along with the SNVs in the repeat and prophage regions of the N16961 genome, which were identified and masked using NUCmer (47) and PHAST (48) for core genome phylogeny. Core SNPs were aligned and used to generate a maximum-likelihood tree using fasttree v2.1.10 (49). The distance matrix was constructed with snp-dists v0.6.2. Clusters were defined based on the SNP threshold of 40 or fewer SNPs.

To estimate the phylodynamics of the Australian and international V. cholerae O1 strains, a temporal analysis using the Bayesian Evolutionary Analysis Sampling Trees (BEAST) version 2.4.7 software package was performed (50). The root-to-tip regression was fitted to elucidate the relationship between sampling time (year) and the expected number of nucleotide substitutions along the tree using Tempest v 1.5.1 (http://tree.bio.ed.ac.uk/) prior to running the Bayesian analysis. A relaxed clock exponential model and the general time-reversible (GTR) gamma nucleotide substitution model were selected based on path sampling and stepping stone sampling methods and the correlation (*R*^2^ = 0.80) between the sampling time and root-to-tip divergence (51, 52). This model was implemented using the Markov chain Monte Carlo method run for 150 million generations and sampled every 10,000 generations. The effective sample sizes were >200 for all estimated parameters. Three independent analyses were performed and combined using LogCombiner v1.10 (53). The tree data were summarized to generate the maximum clade credibility tree using Tree Annotator with first 10% burn-in. These results were visualized using Interactive Tree Of Life (iTOL) version 4 (https://itol.embl.de) (54).

### Data availability.

Raw sequence files and associated metadata have been submitted to the NCBI with BioProject ID PRJNA670297.
